# A Review of Matched-pairs Feature Selection Methods for Gene Expression Data Analysis

**DOI:** 10.1016/j.csbj.2018.02.005

**Published:** 2018-02-25

**Authors:** Sen Liang, Anjun Ma, Sen Yang, Yan Wang, Qin Ma

**Affiliations:** a Key Laboratory of Symbol Computation and Knowledge Engineering of Ministry of Education, College of Computer Science and Technology, Jilin University, Changchun 130012, China; b Bioinformatics and Mathematical Biosciences Lab, Department of Agronomy, Horticulture and Plant Science, Department of Mathematics and Statistics, South Dakota State University, Brookings, SD 57007, USA; c BioSNTR, Brookings, SD, USA

**Keywords:** Matched-pairs feature selection, Matched case-control design, Paired data, Gene expression

## Abstract

With the rapid accumulation of gene expression data from various technologies, e.g., microarray, RNA-sequencing (RNA-seq), and single-cell RNA-seq, it is necessary to carry out dimensional reduction and feature (signature genes) selection in support of making sense out of such high dimensional data. These computational methods significantly facilitate further data analysis and interpretation, such as gene function enrichment analysis, cancer biomarker detection, and drug targeting identification in precision medicine. Although numerous methods have been developed for feature selection in bioinformatics, it is still a challenge to choose the appropriate methods for a specific problem and seek for the most reasonable ranking features. Meanwhile, the paired gene expression data under matched case-control design (MCCD) is becoming increasingly popular, which has often been used in multi-omics integration studies and may increase feature selection efficiency by offsetting similar distributions of confounding features. The appropriate feature selection methods specifically designed for the paired data, which is named as matched-pairs feature selection (MPFS), however, have not been maturely developed in parallel. In this review, we compare the performance of 10 feature-selection methods (eight MPFS methods and two traditional unpaired methods) on two real datasets by applied three classification methods, and analyze the algorithm complexity of these methods through the running of their programs. This review aims to induce and comprehensively present the MPFS in such a way that readers can easily understand its characteristics and get a clue in selecting the appropriate methods for their analyses.

## 1 Introduction

During the last two decades, feature selection techniques have become an active and fruitful research field in machine learning [[1], [2], [3], [4]], pattern recognition [5,6], and bioinformatics [[7], [8], [9]]. Feature selection, a.k.a. Variable selection or gene selection (in bioinformatics), is the process of selecting a subset of relevant features for model construction or interpretation of results. It improves model predictive accuracy and reduces model complexity by eliminating irrelevant and redundant features and provides a better understanding of the underlying processes [10]. Many novel methods have been proposed recently, such as the minimum-Redundancy-Maximum-Relevancy (mRMR) method proposed by Peng et al. which selects features using mutual information as a proxy for computing relevance and redundancy among features [11], and the Max-Relevance-Max-Distance (MRMD) method proposed by Zou et al. that selects features with strong correlation with labeled and lowest redundancy features subset [12]. With the rapid expansion of gene expression data, higher gene dimensionality has been generated in limited samples. The feature selection techniques are playing more and more pivotal roles in high-dimensional data analyses, especially in gene function enrichment analysis, cancer biomarker detection, and drug targeting identification in precision medicine. Recently, Zou et al. proposed a new method to predict TATA-binding proteins with feature selection and dimensionality reduction strategy [13]. Tang et al. proposed novel selection strategies to identify highly tissue-specific CpG sites and then constructed classifiers to predict primary sites of tumors [14].

However, it is still a challenge to choose the appropriate methods for specific problems and retrieve the most reasonable ranking features in gene expression data analysis. Nowadays, using the existing next-generation sequencing techniques, such as microarray and RNA-seq, developed for gene expression profiling, the paired gene expression data under matched case-control design (MCCD) is becoming increasingly popular. Such data has frequently been used in multi-omics studies and may increase the feature selection efficiency by offsetting similar distributions of confounding features [15]. Nevertheless, the appropriate feature selection methods specifically designed for paired data accounting on MCCD, which is so-called matched-pairs feature selection (MPFS), have not been maturely developed in parallel.

There are many popular MPFS methods and strategies for bioinformatics research. Several studies have been managed to account for paired data in their algorithms, which can be categorized into three groups. First, the test statistic uses original and modified paired *t*-test to rank relevant features by evaluating significant levels which is often followed by a classification approach to improve model predictive accuracy. Such kind of methods is comparatively time-consuming and may return a preliminary feature selection results. Second, the conditional logistic regression (CLR) [16] is a modeling approach widely be used in MCCD studies to identify features significantly associated with case-control status. CLR has considerations of the interaction between features and make a better selection results when potential correlations exist. Third, the boosting strategy addresses classification problems with matched case-control responses. In machine learning, boosting is usually combined with many weak classifiers to build a powerful committee. Since Friedman et al. [17] described boosting as a method for the additive model using an exponential loss criterion, researchers employed boosting to identify significant features with paired data within a classifier task [18]. The boosting strategy is more powerful and time-consuming, which always need to be wrapped with a classifier, e.g., support vector machines (SVM) [19].

This review provides a survey of existing MPFS methods and applications for paired gene expression data under MCCD. Two real gene expression datasets from The Cancer Gene Atlas database (TCGA) [20] and Gene Expression Omnibus database (GEO) [21] were selected to evaluate the performance of MPFS methods and traditional unpaired feature selection methods. The rest of the paper is organized as follows: Section 2 introduces the feature selection techniques in general and presents overall classification strategies according to different data properties. In Section 3, the MPFS problem is defined and then the existing MPFS methods are summarized according to the above three feature selection groups. In Section 4, we compare the performance of ten methods, including eight MPFS methods and two traditional unpaired methods on the two real datasets and three classification methods, i.e., SVM, Gaussian Naïve Bayesian (GNB) [22], and Logistic Regression [23]. The running times of these methods are also recorded simultaneously as another vital criterion to help readers select the appropriate method for different environments. We further discuss several challenges for the development of the MPFS techniques and their further applications in many other bioinformatics research fields in Section 5. Finally, the conclusions are clearly drawn in the last section.

## 2 Feature Selection Techniques

The most acceptable benefit of feature selection is to help improving accuracy and reducing model complexity, as it can remove redundant and irrelevant features to reduce the input dimensionality and help biologists identify the underlying mechanism that connects gene expression with diseases or interested phenotype.

Feature selection techniques have been successfully applied in many real-world applications, such as large-scale biological data analysis [[24], [25], [26]], text classification [27], information retrieval [28], near-infrared spectroscopy [29], mass spectroscopy data analysis [30], drug design [31,32], and especially the quantitative structure-activity relationship (QSAR) modeling [33,34]. In cancer research community, feature selection has also been widely applied in different omics data analyses: mRNA data [9,35], miRNA data [36,37], whole exome sequencing data [38], DNA-methylation data [39,40], and proteomics data [41,42]. Recently, some researchers have applied feature selection techniques on integrative analysis of multi-omics data. Chen et al. reviewed multivariate dimension reduction approaches which can be applied to the integrative exploratory analysis of multi-omics data [43]; Mallik et al. developed a new feature selection framework for identifying statistically significant epigenetic biomarkers using maximal-relevance and minimal-redundancy criterion based on multi-omics dataset [44]; and Liu et al. [45] developed two methods based on the proportional hazards regression [46], named SKI-Cox and wLASSO-Cox approaches, to perform feature selection on different multi-omics datasets.

### 2.1 Unpaired Feature Selection Methods

It is not trivial to choose the appropriate feature selection method for a given scenario, hence, several classification strategies of unpaired feature selection techniques have been approached. The most widely-used classification strategy classified the methods into the filter, wrapper and embedded, based on the integrated classifiers [7,10,47]. The filter approach separates feature selection from classifier construction and assesses the relevance of features only relying on the intrinsic properties of data [48,49], which have frequently been used in high dimensional data analysis (e.g., microarray data). The wrapper approach evaluates classification performance of selected features and keeps searching/optimizing until certain accuracy criterion is satisfied [50,51]. The embedded approach embeds feature selection within classifier constructions to perform less computationally intensive than wrapper methods [52,53] and has the advantage to interact with the classification models [47]. Except for utilizing each feature selection method individually, the ensemble feature selection has come up by integrating multiple methods into one algorithm. It has the most prominent advantageous ability to handle stability issues which are usually poor in the existing feature selection methods, under the assumption that the output of multi-model is better than any individual model [54].

Besides, various taxonomies for feature selection are also developed. Depending on whether the original features are transformed into new features, the terminology “feature extraction” is specifically defined from the feature selection technologies [55]. Furthermore, feature selection can also be divided into univariate and multivariate types, based on feature independence [8]. With the search optimal feature perspectives, Wang et al. formulated feature selection as a combinatorial optimization or search problem, and categorized the methods into exhaustive search, heuristic search, and hybrid method [56].

### 2.2 A Different Perspective of Feature Selection By Data Properties

Recently, some researchers began to consider the data properties in developing or choosing appropriate feature selection methods. Ang et al. observed the gene expression data can be fully labeled, unlabeled, or partially labeled [57]. With such a fact, they correspondingly separated feature selection methods into three categories: supervised, unsupervised and semi-supervised. Tan et al. found the popular MCCD in microarray experiments lacked appropriate feature selection method. To solve the problem, they proposed a method based on modified *t*-statistic in their study [58]. From then on, many researchers began to develop new feature selection methods for paired data under MCCD [18,[59], [60], [61], [62], [63], [64], [65]]. Additionally, the paired gene expression data under MCCD is often referred to obtain two gene expression profiles from case tissues and control tissues, respectively. In cancer research study, case tissue often relates to tumor tissue and control tissue is the corresponding adjacent non-tumor tissue.

## 3 Matched-pairs Feature Selection

### 3.1 Problem Description

Before we survey the feature selection methods on paired data, it is worthwhile to give descriptions of MPFS problems and the corresponding goals.

Considering *n* Npaired data samples for *X* = {*x*_*i*_| *i* = 1, 2, …, *n*} under 1 : *m* MCCD. *p* and *q* are used to represent the number of case experiments and control experiments, respectively, where *q* = *mp*. For each paired data *i*, there is *X*_*i*_ = {*x*_*ij*_| *j* = 1, 2, …, *p* + *q*}, and let *Z*_*i*_ denotes the case-control status of *X*_*i*_ with *Z*_*i*_ = {*z*_*ij*_| *j* = 1, 2, …, *p* + *q*}, such that *Z*_*ij*_ = 1 for case and 0 for control. Given each sample *K* features, as *L* = (*l*_*k*_| *k* = 1, 2, …, *K*), we denote *X*_*ij*_ = (*x*_*ijk*_| *k* = 1, 2, …, *K*); as the vector data with *K* features of the *i*^*th*^ paired data under the *j*^*th*^ paired element. The aim of MPFS method is to find out the optimal subset features from all *K* features, account on the 1 : *m* MCCD.

**Fig. 1:**
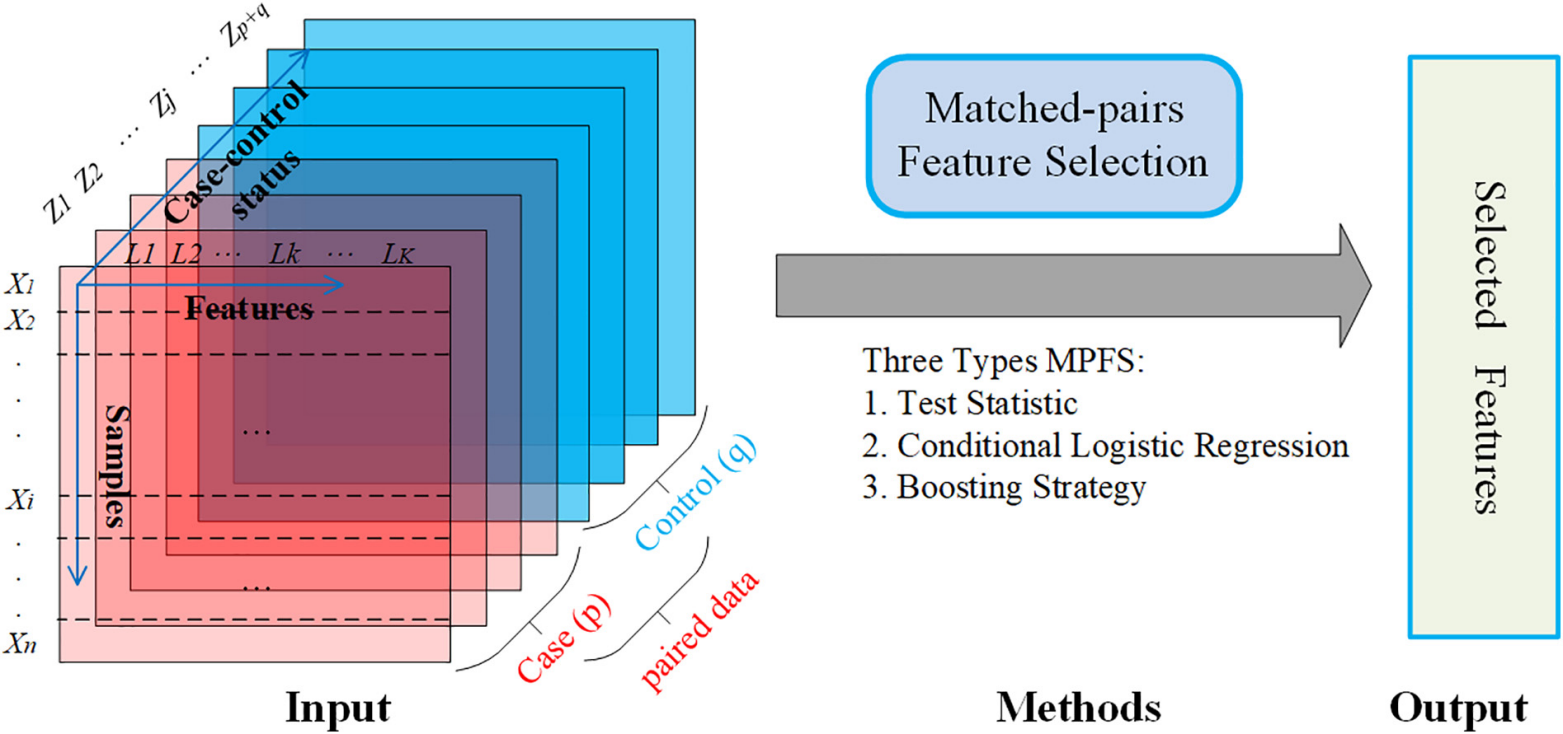
Matched-pairs feature selection problem description. Paired data with matched *p* cases and *q* controls as input for the MPFS method and getting selected features as output.

Recently, almost all algorithms were developed under 1:1 MCCD, as data are paired and easy analysis, where *m* = 1 so that *p* = *q*, *X*_*i*_ = (*X*_*i* 1_, …, *X*_*i* *p*_, *X*_*i* *p*+1_, …, *X*_*i* *p*+*q*_) and *Z*_*i*_ = (*Z*_*i* 1_, …, *Z*_*i* *p*_, *Z*_*i* *p*+1_, …, *Z*_*i* *p*+*q*_). In paired gene expression data, *p* and *q* often equal to 1, so that *X*_*i*_ = (*X*_*i*1_, *X*_*i*2_). In Fig. 1, we illustrate the matched-pairs features problem with matched *p* cases and *q* controls.

### 3.2 Methods Survey

As mentioned, depending on the underlying methods, MPFS approaches can be divided into three categories: test statistic, CLR, and boosting strategy (Table 1). Here we surveyed most of the MPFS methods from literature and discussed each one in detail.

**Table 1:** Matched-pairs feature selection survey. This table lists the matched-pairs feature selection methods in this article with its method name (second column), software (third column) and literature (fourth column) through three groups: test statistic, CLR, and boosting strategy.

	Method	Software^a^	Literature
Test statistic	Paired *t*-test	R package “PairedData”	Hsu et al. [66]
	Modified paired *t*-test	–	Tan et al. [58]
	Fold-change paired *t*-test	–	Cao et al. [62]
Conditional logistic regression	RP-CLR	R package “RPCLR”	Balasubramanian et al. [64]
	PCU-CLR	R package “penalized”	Qian et al. [15]
	BVS-CLR	R package “coda”	Asafu-Adjei et al. [65]
Boosting strategy	WL2Boost	Source code in paper	Adewale et al. [18]
	1-step PQLBoost	–	Adewale et al. [18]

a Using “–” if no specific software found for the method.

#### 3.2.1 Test Statistic for MPFS

Test statistic methods are widely used in testing if two groups data obey one distribution, which has a low computational complexity and is easy to carry out. Paired *t*-test methods are suite for paired data, especially in gene expression analysis [66,67]. Modified paired *t*-test method and fold-change paired *t*-test method are more adapted to MCCD settings.

##### 3.2.1.1 Paired *t*-Test

The original statistic method of paired *t*-test [66,67] has been widely used in paired data analysis, especially in identifying differential gene expression. Given 1:1 matched case-control setting, where *Z* = (1, 0), the difference between paired case and control *X* with the *k*^*th*^ feature is given

(1) $$d_{i,k}=X_{i,1,k}-X_{i,2,k}$$

For all *n* samples, The mean difference ${\bar{d}}_{k}$ with the *k*^*th*^ feature can be given by ${\bar{d}}_{k}=\left(1/n\right)\sum_{i=1}^{n}d_{i,k}$, and the standard error of d under the *k*^*th*^ feature is $s_{k}=\sqrt{\sum_{i=1}^{n}{\left(d_{i,k}-{\bar{d}}_{k}\right)}^{2}/\left(n-1\right)}$. Combining ${\bar{d}}_{k}$ and *s*_*k*_, the paired *t*-test statistic for the *k*^*th*^ feature is defined as

(2) $$t_{i}={\bar{d}}_{i}/s_{i}$$

With each feature's statistic and its corresponding *p-value*, we can rank it and make the feature selection analysis.

##### 3.2.1.2 Modified Paired *t*-Test

Tan et al. developed a modified paired *t*-test statistic to identify a subset of relevant features that served as a basis for classification via support vector machines (SVM) [58]. The gene and feature selection were optimized by setting thresholds in a leaving one-pair out cross-validation procedure using SVM [68].

In this method, the authors added a positive constant *s*_0_ to the denominator of Eq. (2) to induce a modified paired *t*-test statistic, denoted as *t*_*k*_^'^ and shown as:

(3) $$t_{k}^{'}={\bar{d}}_{k}/\left(s_{k}+s_{0}\right)=t_{k}\left(1/\left(1+s_{0}/s_{k}\right)\right)$$

According to a study of Tibshirani et al. [69], *s*_0_ is the median of *s*_*k*_. They also specified a threshold Δ for selecting features with the condition of |*t*_*k*_^'^| − Δ > 0, and obtained the optimal subset features through a leaving one-pair out cross-validation.

##### 3.2.1.3 Fold-change Paired *t*-Test

Cao et al. proposed another modified version of paired *t*-test statistic using the fold-change value instead of *d*_*i*, *k*_ between case and control samples in Eq. (1) [62]. They utilized *q-value* in the False Discovery Rate method [70] to measure statistical significance for each feature.

The author hypothesized that different paired data have different experimental environments and conditions. It is believed that the measurement of the difference between case and control in originally paired *t*-test is unstable and lack of enough generalization ability among different data sets. To address such problem, they used the fold-change value between case and control to replace Eq. (1), which is given by

(4) $$d_{i,k}=\left\{\begin{array}{c} {\mathrm{FC}}_{i,k}-1\;\left({\mathrm{FC}}_{i,k}\geq 1\right)\\ 1-1/{\mathrm{FC}}_{i,k}\;\left({\mathrm{FC}}_{i,k}<1\right) \end{array}\right)$$

where the fold-change value *FC*_*i*, *k*_ equals to *X*_*i*1, *k*_/*X*_*i*2, *k*_.

#### 3.2.2 Conditional Logistic Regression for MPFS

In matched-pairs studies, the standard analytical approach uses CLR to identify features significantly associated with case-control status [71]. A CLR model is a specialized logistic regression that allows users to consider stratification and matching, which are usually employed to investigate the relationship between case and control data. However, with dramatically increasing data dimension, CLR strategy becomes computationally intensive, and model convergence problems are foreseeable [65]. So far, several new feature selection algorithms have been developed to solve the issue and are presented as follows.

##### 3.2.2.1 Random Penalized Conditional Logistic Regression (RP-CLR)

Balasubramanian et al. proposed an RP-CLR method to assess variable importance associated with matched case-control status in high dimensional data setting [64]. The algorithm is based on penalized conditional likelihood model for adjusting for the matched case-control design and accounting the two-way interaction among features and incorporates some attractive characteristics in the random forest to assess variable importance. The method is proposed for 1:1 matched studies and can be generalized to 1:m matched studies. Specifically, the algorithm contains three steps: (i) bootstrap *M* paired datasets to form the original paired data set; (ii) for each bootstrap paired data set, a random subset of *K* features are selected to fit a conditional logistic model with penalty, and the significance of each feature is assessed; and (iii) the average variable significance score in overall *M* bootstrap is calculated for users to achieve the goal of feature selection.

##### 3.2.2.2 Penalized Conditional and Unconditional Logistic Regression (PCU-CLR)

Qian et al. presented a two-stage procedure, based on penalized conditional and unconditional logistic regression approaches, to tackle the dual goals of variable selection and prediction problems under MCCD [15]. In the first stage, variable selection is carried out to estimate regression coefficients *β* by using the penalized log-likelihood as

(5) $$\log\left(l_{C}\left(\beta \right)\right)-\sum_{i=1}^{p}g_{\lambda 1}\left(\left|\beta_{i}\right|\right)-\sum_{i=1}^{p}\sum_{j>i}^{p}g_{\lambda 2}\left(\left|\beta_{\mathrm{ij}}\right|\right)$$

where *log*(*l*_*C*_(*β*)) is the log conditional likelihood function of *β*. *g*_*λ*1_(·) and *g*_*λ*2_(·) are penalty functions for variables and two-way interactions, respectively. To select the optimal penalty parameters, *λ*1 and *λ*2, ten-fold cross-validation method is employed in the model [72]. At last, variable selection stage can be completed by maximizing the likelihood function (Eq. (5)). In the second stage, estimated *β* can be used to fit an unconditional logistic regression model with matched case-control data for prediction.

##### 3.2.2.3 Bayesian Variable Selection Conditional Logistic Regression (BVS-CLR)

Compared to penalized methods on a CLR model, the Bayesian method has more advantages in feature selection, as it provides exact inference and a natural way of combining prior information with data. Penalized methods select features by determining coefficient estimates only in non-zero models, yet in Bayesian methods, more information is provided by offering coefficient estimates and giving probability estimates for each feature. Combining the key benefits of the Bayesian method and CLR for feature selection technique, Asafu-Adjei et al. proposed a new approach that formulated Bayesian variable selection (BVS) in a CLR framework, called BVS-CLR [65]. Although this method mainly focuses on 1:1 case-control matching, Asafu-Adjei claimed that it could indeed handle more general cases of 1 : *m* matching. The simple description of the approach is shown below.

Considering the 1:1 matched case-control setting, in the first place, the likelihood function is specified based on a CLR model. The conditional log-likelihood function is given by

(6) $$l_{C}\left(\beta \right)=\log\left(\prod_{i=1}^{N}p_{i1}^{Z_{i1}}\right)$$

where the coefficient vector *β* = (*β*_1_, …, *β*_*K*_) so that *β*_*k*_ denotes the coefficient for feature *L*_*k*_. *p*_*i*1_ is the probability that the first member of pair *i* is a case. Given (*X*_*i*1_, *X*_*i*2_) and *Z*_*i*1_ + *Z*_*i*2_ = 1, *p*_*i*1_ is defined as

(7) $$p_{i1}=P\left(Z_{i1}=1|Z_{i1}+Z_{i2}=1,X_{i1},X_{i2}\right)={\left\{1+\exp\left[-\sum_{1}^{K}\beta_{k}\left(X_{i1,k}-X_{i2,k}\right)\right]\right\}}^{-1}$$

Next, by applying the Bayesian method, the posterior distribution of *γ* and *β* can be obtained, where *γ* = (*γ*_1_, …, *γ*_*K*_) is a binary vector to denote whether the features are retained or not. Let *γ*_*k*_ equals 1 for retained feature *k*, and 0 otherwise. Given the prior distribution of *β* and *γ* as *π*(*β*| *γ*)  and *π*(*γ*), respectively, the posterior distribution is given by

(8) $$p\left(\beta ,\gamma |X,Z\right)\;\propto l_{C}\left(\beta \right)\times \pi \left(\beta |\gamma \right)\times \pi \left(\gamma \right)$$

At last, Markov chain Monte Carlo (MCMC) [73] sampling via the Metropolis-Hastings (MH) [74] algorithm is used to estimate the posterior distribution of Eq. (8). After MCMC sampling and iterations, the sequence {(*β*^[1]^, *γ*^[1]^), …, (*β*^[*S*]^, *γ*^[*S*]^)} can be obtained from each iteration. Employed with MH algorithms, they estimated the posterior inclusion probabilities *p*(*γ*_*k*_ = 1| *X*, *Z*) and the coefficients *β*_*k*_, which can be used to rank features and determine the optimal models.

#### 3.2.3 Boosting Strategy for MPFS

Boosting is another successful strategy for high-dimensional feature selection. Adewale et al. developed two modified boosting methods for correlated binary response data [18].

##### 3.2.3.1 Boosting Weighted L_2_ Loss (WL_2_Boost)

The first method based on the functional gradient decent boosting was dubbed “WL_2_Boost” [75,76]. The loss function adopts to the L_2_ loss if the weights are taken to be an identity matrix. The weight matrix represents the unknown variance-covariance matrix of response. Compared to the standard functional gradient descent approach, the loss function is modified by updating the variance-covariance matrix as the boosting iteration progresses.

##### 3.2.3.2 1-Step Penalized Quasi-Likelihood (1-Step PQLBoost)

The second method is called 1-step PQLBoost, which modifies the likelihood optimization boosting algorithm via a generalized linear mixed modeling approach, described by Friedman et al. [17] and Tutz et al. [77]. It is similar to the penalized quasi-likelihood (PQL) approach, and its numerical approximation of integrals can be achieved via fitting linear mixed models (random intercept) to pseudo-responses. In the implementation, the authors employed a one-step fitting instead of iterative fitting of linear mixed models in PQL. Therefore, they dubbed this method as one-step penalized quasi-likelihood boosting (1-step PQLBoost). After the model classifier ${\hat{F}}_{M}\left(X\right)$ is obtained from both methods, the relative influence of each feature in the boosting procedure can be calculated via the following influence measurement [75]:

(9) $$I_{l}={\left(E\left[\partial F\left(X\right)/\partial x_{l}\right]\;/\mathrm{var}\left(x_{l}\right)\right)}^{1/2},\;l=1\ldots p$$

Above all, we have described three groups MPFS methods: test statistic, conditional logistic regression, and boosting strategy. The test statistic methods use original and modified paired *t*-test to rank relevant features and are often followed by a classification approach to improve model predictive accuracy. The conditional logistic regression methods are widely used in MCCD studies to identify features significantly associated with case-control status and have taken the interaction between features into consideration. The boosting strategy addresses classification problems with matched case-control responses.

## 4 Experimental Validation

To compare the performance of the above-mentioned eight MPFS methods and two traditional unpaired feature selection methods (mRMR and MRMD [12]), two breast cancer gene expression datasets were extracted from the TCGA [20] and GEO [21] databases and three classification methods [23] were applied for the following experiments.

Both datasets contain gene information from tumor tissue and matched-pair normal tissue. The TCGA-BRCA dataset, downloaded from TCGA, contains 113 samples of case-control patients, and the GSE70947 dataset, downloaded from GEO, contains 143 samples of case-control patients. The experiments include three main steps: (i) data pre-processing and normalization, (ii) generalization of gene significance ranking list for each method, and (iii) comparison of the performance of all ten methods by applying three classification methods based on the generated ranking lists.

The two datasets have been pre-analyzed by the following processes: (i) Merging different probes of the same gene by selecting the maximum value to present the gene expression level; (ii) Substitution of missing value is performed using the mean of the expression values, once only <1% missing data exists. Otherwise, such a gene will be discarded; (iii) Normalizing the two datasets by scaling to 0–1; and (iv) Filtering genes by *p*-value < 0.005 (*t*-test), variance >0.1, and the absolute fold-change >0.5 between case and control data.

After the above pre-processing steps for the case and control data matrix, the ten feature selection methods are implemented to both datasets to obtain gene ranking lists. The lists were then integrated into a classifier to obtain the accuracy curves by ten-fold cross-validation [72], which compares the performance of each feature selection method to assess their effectiveness and stability. Here we used three classifiers to validate the performance of ten methods: SVM, Gaussian Naive Bayesian, and Logistic Regression.

**Fig. 2:**
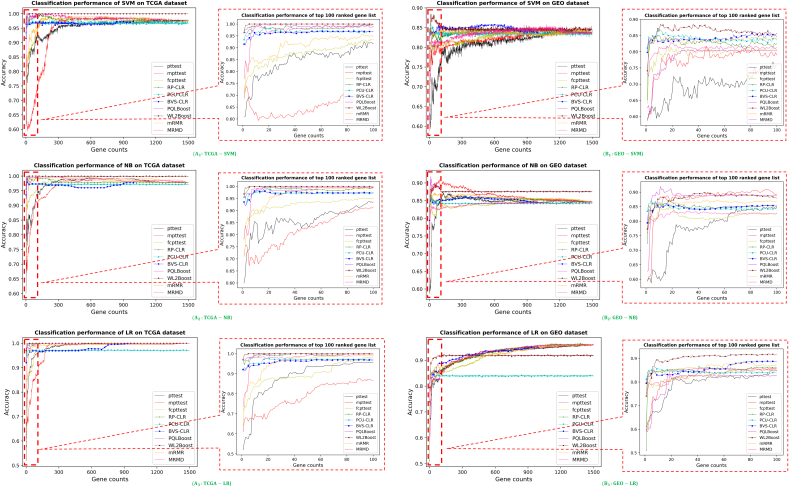
Performances of the ten methods on two datasets. Fig. (A1–A3) are the classification performance of each method with top 1500 ranked gene list on TCGA dataset, and Fig. (B1–B3) are on GEO dataset. Fig. A1–B1, A2–B2, and A3–B3 are the comparison of SVM, GNB and Logistic Regression (LR) methods for both datasets, respectively. Each figure includes performance comparing the result of top 1500 ranked gene list, and a zoomed-in figure indicating the detail the of the top 100 ranked gene list. The accuracy data of PQLBoost and BVS-CLR methods are omitted after 1000 gene counts due to the need of enormous running time (exceeding 48 h).

The accuracy curves of the top 1500 genes in each method are shown in Fig. 2. The results showed that WL2Boot method has the highest accuracy and most stable performance among all the ten methods and two gene lists; and PQLBoost was also competitive but showed less satisfied accuracy compare to WL2Boot. Meanwhile, the three types of *t*-test methods, pttest, mpttest and fcpttest performed less satisfied as they only identify differential genes when the case data and control data are obeying to the same distribution without additional feature information. The performances of the three conditional logistic regression methods, PCU-CLR, RP-CLU and BVS-CLR, and one classic unpaired method, MRMD, were shown moderate for both small gene counts (100) and large gene counts (1500), while mRMR was only better than the *t*-test methods. All the ten methods showed great accuracy higher than 0.85 with gene counts grew, except for SVM-GEO. Additionally, in both datasets, the ten methods showed unsatisfied or slow-growing accuracy for SVM classifier at the lower gene counts. As a result, under the matched-pairs data setting, most MPFS methods, except the modified *t*-test methods, are the better choices for feature selection tasks than traditional unpaired feature selection methods.

**Fig. 3:**
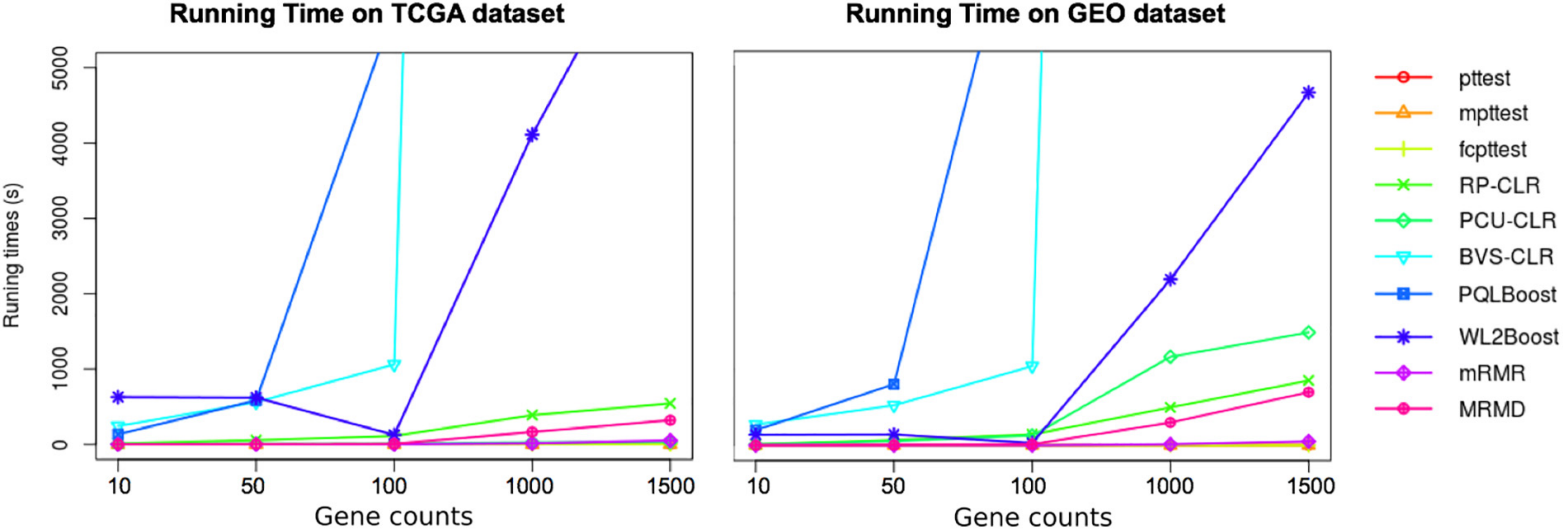
Comparison of running time. It should be noted that the running time is the time for producing the gene lists for each method. Left figure is the comparison of ten methods on TCGA dataset, and right figure is on GEO dataset.

On the other hand, running time is also a crucial indicator to evaluate the performance of methods. Here, we only record the running times of generation of gene list with specific gene counts, 10, 50, 100, 1000 and 1500 (Fig. 3), as the executing time for accuracy validation is almost the same among three classification methods. In both datasets, more time was required for 1-Step PQLBoost, BVS-CLR, and WL2Boost methods compared with the other seven. Moreover, more running time was needed for higher gene counts for all ten methods. Combining with the accuracy results, we concluded that (i) WL2Boost method is the optimized method with high accuracy and low running time when the gene count is low; (ii) PCU-CLR and RP-CLR show higher tradeoff for higher gene counts, with acceptable running time and high accuracy compared to the other methods; (iii) Though BVS-CLR and PQLBoost also show satisfied accuracy performances, their running times are unacceptable, and are not recommended for normal feature selection; and (iv) the three modified *t*-test methods are suitable for high gene counts analysis, since their accuracy have no significant difference and required the least running time compared to other methods.

## 5 Discussion

This paper presented a review of current matched-pairs feature selection (MPFS) methods for paired gene expression data. With a description of feature selection application and MPFS problem, we reviewed the current approaches of MPFS through three categories, i.e., test statistic, CLR, and boosting strategy. Differ from the commonly categorized feature selection approaches (filter, wrapper, and embedded), we dealt feature selection with gene expression data as unpaired and MPFS methods by considering MCCD or not.

**Fig. 4:**
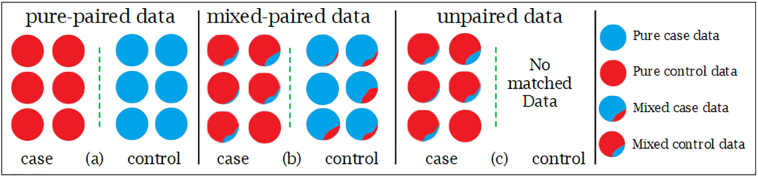
Paired and unpaired data diagram. Three data types for feature selection: (a) pure-paired data type, which has pure case and control data; (b) mixed-paired data type, which has different mixing degree of mixture case and control data, (c) unpaired data type, which contains mixture case data without matched control data. It is noteworthy that the mixing degree is referred to the ratio between control part (blue) and case part (red) on one case sample, and vice versa on a control sample.

The paired data can be divided into pure-paired data and mixed-paired data under MCCD, and the mixed-paired data is regarded as pure-paired to reduce the model complexity and minimize the mixing effect. However, the unpaired data, which contains mixture case data without matched data, is usually obtained when matched data is missing or MCCD experiment is not performed. In Fig. 4, we illustrate the differences among the three pair types. In the sequencing transcriptomic data, such as microarray and RNA-seq, the formation of tumor tissue is a mixture of more tumor cells (cases) and few non-tumor cells (controls), while the adjacent non-tumor tissue contains more non-tumor cells (controls) and few tumor cells (cases). In this case, we denote the paired data as mixed-paired data. To address the mixing degree, TCGA project [78] uses the property of normal cells percentage based on the tumor tissue image. However, with the up-to-date RNA-seq technique, we can get gene expression profile for every single tumor or normal cell on cell resolution level, described as pure-paired data whose case and control data are not mixed at all.

The originally paired *t*-test is most commonly used in practical paired gene expression data analysis, as it is easy to implement and very efficient. The modifications of paired *t*-test methods have higher sensitivity and specificity than the original. However, they only involve univariate tests, which do not control the effects of other features and can lead to the fallacious identification of relevant features. The CLR model is a standard and effective analytical approach to significantly identify features associated with case-control status yet with higher computational intensity and convergence problems. To solve the issue, Balasubramanian et al. designed the RPCLR algorithm [64], and Qian et al. designed a two-stage procedure based on penalized conditional and unconditional logistic regression approaches [15]. Moreover, Asafu-Adjei et al. proposed the BVS-CLR method [65] to provide more information by offering coefficient estimates and giving probability estimates for each feature, while it may remain problems with selection accuracy when the correlation between features increases. Boosting strategy feature selection approaches successfully dealt high-dimensional data, as it can combine with many weak classifiers to build a powerful committee. Adewale et al.'s two variant versions of boosting algorithm [18] focused on high-dimensional data with correlated binary outcomes, but may also have troubles in identifying interactions when dealing with different features or small sample sizes data.

MPFS can be widely applied in bioinformatics, e.g., gene function enrichment analyses, cancer biomarker detection, drug targeting identification, etc. To be specific, here are several examples: (i) Identifying important CpG sites. CpG site refers to a double-stranded sequence where cytosine and guanine are separated by only one phosphate, and gene expression can be altered by cytosine methylation on that site. Sun et al. [60] selected important methylated CpG sites between ovarian cancer cases and healthy controls using DNA methylation data. (ii) Identifying clinical risk features for diseases. Scott et al. [79] used matched case-control study to clinical exam features for utility optimization to identify the risks of early transition from depression to bipolar disorders in youth; and Giuliano et al. [80] studied the effect of age, sex and clinical features on the volume of Corpus Callosum in preschoolers with Autism Spectrum Disorder using case-control study. (iii) Biomarker discovery. Xu et al. [81], Anglim et al. [82] and Tsou et al. [83] have reported the results of cancer biomarker discovery using MCCS; and Zak et al. [84] discovered a blood RNA signature related to tuberculosis disease by comparing data from participants who developed active tuberculosis disease (progresses) and those who remained healthy (matched controls). (iv) Image biomarkers discovery. Kloppel et al. [85] described an investigation involving a matched design to discover imaging biomarkers for Alzheimer's disease. (v) Identifying drug targets. Gronich et al. [86] evaluated the association between tyrosine kinase-targeting drugs and the risk of new-onset heart failure, using nested case-control analysis. (vi) Clinical supplementary diagnosis. By comparing several predicted models, Holsbø et al. [87] proposed a biologically motivated variable selection scheme for predicting breast cancer metastasis based on the assumption that gene expression intensity, as a function of time, should be diverged between cases and controls.

Although numbers of researchers have explored MPFS with numerous methods, challenges are still ahead of us. First of all, as discussed in Section 2, the paired data can be divided into either mixed-paired data or pure-paired data. To our best knowledge, insufficient studies have been developed for such differentiation in gene expression data analysis. Meanwhile, the mix-paired data from RNA-seq and microarray is always regarded as pure-paired data. Considering the involvement of mixing the degree of paired data in MPFS, it may be a direction with quite a developmental potentiality in the future. Furthermore, no study has been carried out to purpose feature selection methods for pure Single-Cell paired data. Another promising direction for MPFS is to develop hybrid and ensemble frameworks to enhance the robustness of selected feature subsets. Beatriz et al. reviewed [88] the evolutional computation on feature selection and suggested that more attentions should be given to the issue of robustness of the feature selection methods.

Besides that, the stability of gene selection is also extremely important in bioinformatics [[89], [90], [91]]. To this end, the research of stability of feature selection can be split into two categories: stability testing & measurement and method devisal for stability improvement. For testing and measurement, a lot of merits have already been developed, such as cross-validation [92], bootstrapping [93], and fixed overlap partitioning. To improve the stability, the most popular idea is using the ensemble method. However, the method for stability improvement of MPFS under MCCD is still needed.

The last challenge, as another interesting future direction, is to integrate two or more omics data using MPFS in cancer research. Chen et al. reviewed multivariate dimensional reduction approaches that can be applied to the integrative exploratory analysis of multi-omics data [43]. Mallik et al. developed a new framework for identifying statistically significant epigenetic biomarkers using the maximal-relevance and minimal-redundancy criterion based feature selection for multi-omics dataset [44]. Liu et al. developed two methods based on the proportional hazards regression, named SKI-Cox and wLASSO-Cox, to perform feature selection on different omics datasets [45].

Besides the challenges discussed above, other issues on feature selection methods still exist, as the same as MPFS approaches, such as the problem of small sample size in big dimensional data sets, data class imbalance, computational complexity, especially for the conditional logistical regression model, and the assessment of MPFS.

## 6 Conclusion

In this review, we recalled the concepts of feature selection techniques and focused on MPFS methods for gene expression data analysis. We classified the existing algorithms into three groups: test statistic, CLR, and boosting strategy, and evaluated the performance using two breast cancer datasets. From the experimental results of 10 methods on two datasets with three classifiers, we concluded that (1) WL2Boost method may get the best performance when the feature list is not too big, and the users do not care about the running time; and (2) RP-CLR and PCU-CLR methods may get a better tradeoff between high dimensional features and time consuming. At last, we discussed some challenges and exciting directions for the development of MPFS. It is worth noting that, most of algorithms have been proposed in recent years were dedicating to address the feature selection problem associated with the paired data. Based on the development of gene expression profiling technique and the extensive use of MCCD, MPFS approach is a promising technique in the bioinformatics and machine learning cross-field in future.

## Conflict of Interest

The authors claim no conflict of interest.
